# MERS-CoV Accessory ORFs Play Key Role for Infection and Pathogenesis

**DOI:** 10.1128/mBio.00665-17

**Published:** 2017-08-22

**Authors:** Vineet D. Menachery, Hugh D. Mitchell, Adam S. Cockrell, Lisa E. Gralinski, Boyd L. Yount, Rachel L. Graham, Eileen T. McAnarney, Madeline G. Douglas, Trevor Scobey, Anne Beall, Kenneth Dinnon, Jacob F. Kocher, Andrew E. Hale, Kelly G. Stratton, Katrina M. Waters, Ralph S. Baric

**Affiliations:** a Department of Microbiology and Immunology, University of Texas Medical Branch, Galveston, Texas, USA; b Department of Epidemiology, University of North Carolina at Chapel Hill, Chapel Hill, North Carolina, USA; c Department of Microbiology and Immunology, University of North Carolina at Chapel Hill, Chapel Hill, North Carolina, USA; d Pacific Northwest National Laboratory, Richland, Washington, USA; Columbia University College of Physicians & Surgeons

**Keywords:** coronavirus, MERS-CoV, SARS-CoV, live vector vaccines, reverse genetics

## Abstract

While dispensable for viral replication, coronavirus (CoV) accessory open reading frame (ORF) proteins often play critical roles during infection and pathogenesis. Utilizing a previously generated mutant, we demonstrate that the absence of all four Middle East respiratory syndrome CoV (MERS-CoV) accessory ORFs (deletion of ORF3, -4a, -4b, and -5 [dORF3-5]) has major implications for viral replication and pathogenesis. Importantly, attenuation of the dORF3-5 mutant is primarily driven by dysregulated host responses, including disrupted cell processes, augmented interferon (IFN) pathway activation, and robust inflammation. *In vitro* replication attenuation also extends to *in vivo* models, allowing use of dORF3-5 as a live attenuated vaccine platform. Finally, examination of ORF5 implicates a partial role in modulation of NF-κB-mediated inflammation. Together, the results demonstrate the importance of MERS-CoV accessory ORFs for pathogenesis and highlight them as potential targets for surveillance and therapeutic treatments moving forward.

## IMPORTANCE

The initial emergence and periodic outbreaks of MERS-CoV highlight a continuing threat posed by zoonotic pathogens to global public health. In these studies, mutant virus generation demonstrates the necessity of accessory ORFs in regard to MERS-CoV infection and pathogenesis. With this in mind, accessory ORF functions can be targeted for both therapeutic and vaccine treatments in response to MERS-CoV and related group 2C coronaviruses. In addition, disruption of accessory ORFs in parallel may offer a rapid response platform to attenuation of future emergent strains based on both SARS- and MERS-CoV accessory ORF mutants.

## INTRODUCTION

The emergence of Middle East respiratory syndrome coronavirus (MERS-CoV) in 2012 rekindled memories of the severe acute respiratory syndrome (SARS) outbreak at the beginning of the 21st century (1). Characterized by severe respiratory infection and high mortality rates, the novel group 2C coronavirus has produced periodic outbreaks over the past 5 years, leading to 1,936 cases and 690 deaths in 27 countries (2). Importantly, strong evidence links the emergence of these viruses to both camel and bat species, highlighting an ongoing threat posed by MERS-CoV for future outbreaks (3–6). In addition, recent metagenomic analysis has highlighted an expanding breadth of CoVs circulating in animal populations around the world (3, 7–9). Several of these strains have been explored for their capacity to infect human cells, cause disease *in vivo*, and potentially seed future outbreaks (10, 11). Together, these factors highlight the importance of both understanding CoV pathogenesis and identifying platform approaches for therapeutic and vaccine development.

While MERS-CoV and SARS-CoV share similarities in terms of pathogenesis and disease etiology, the molecular mechanisms that they employ during infection varies. For example, MERS-CoV has been shown to be significantly more susceptible to type I interferon (IFN) treatment than SARS-CoV (12–14); however, work from our group suggests that MERS-CoV has greater capacity to modulate downstream interferon-stimulated gene (ISG) responses (15, 16). In addition, SARS-CoV and MERS-CoV share no sequence homology in the context of their accessory open reading frame (ORF) proteins, suggesting differences in immune modulation between the related viruses. Importantly, previous work by our group had highlighted roles for SARS-CoV accessory ORFs in robust infection (17). Similarly, the roles of accessory ORFs 4a and 4b in modulating aspects of the host response suggest a similar need during MERS-CoV infection (18, 19). However, the majority of these MERS-CoV studies occurred outside the context of virus infection and do not address their role in pathogenesis.

Having previously described the generation of mutants lacking all 4 accessory ORFs (dORF3-5 [20, 21]), we set out to evaluate the impact the absence of these viral proteins on MERS-CoV infection in respiratory cells and pathogenesis *in vivo*. Our results suggested that attenuation of the MERS dORF3-5 mutant virus was primarily driven by host responses rather than replication defects. Infection with the accessory ORF mutant disrupted cell processes, augmented IFN responses, and stimulated robust inflammation. Importantly, utilizing a new mouse model of MERS-CoV pathogenesis, the dORF3-5 mutant had robust attenuation in terms of replication as well as pathogenesis. In addition, using the mutant as a live attenuated vaccine provided protection against lethal challenge. Finally, removal of ORF5 implicated its role in modulation of NF-κB-induced inflammation. Together, the results demonstrate the importance of accessory ORFs to MERS pathogenesis and suggest targeting these proteins in parallel may be a viable therapeutic approach for emergent CoV strains.

## RESULTS

### MERS accessory ORF mutant attenuated in human airway cells.

**FIG 1:**
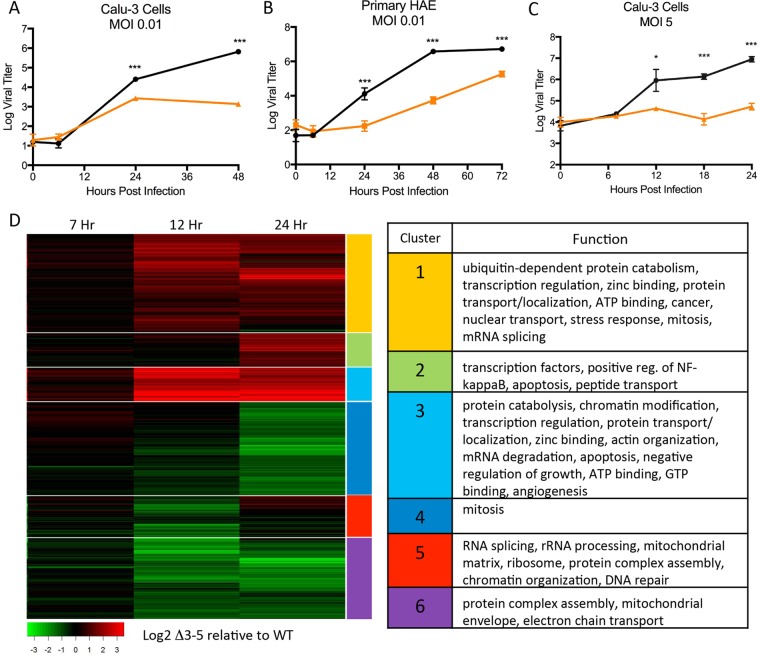
dORF3-5 attenuation mediated by host responses. (A and B) Calu-3 2B4 cells (A) and primary human airway epithelial cells (B) infected with wild-type MERS-CoV (black) or the dORF3-5 mutant (orange) at an MOI of 0.01 and monitored over the study time course. (C) Calu-3 2B4 cells infected at an MOI of 5. (D) RNA expression of the dORF3-5 mutant relative to wild-type virus infection. Clusters are defined by functional category and identified by differential expression score. *P* values are representative of Student’s *t* test: *, *P* < 0.05; ***, *P* < 0.001.

Using our established reverse-genetics system (20), we had previously reported the generation and attenuation of a MERS-CoV mutant virus lacking accessory ORF proteins 3, 4a, 4b, and 5 (dORF3-5). To extend these finding, Calu-3 cells, an immunocompetent human airway respiratory cell line, were infected with wild-type (WT) and dORF3-5 mutant MERS-CoV at a low multiplicity of infection (MOI) (Fig. 1A). While WT virus produced robust viral replication, the dORF3-5 mutant was significantly attenuated. In addition, infection of primary human airway epithelial (HAE) cultures indicated robust attenuation of the dORF3-5 mutant relative to control virus (Fig. 1B). To determine if attenuation is governed by a replication/fitness defect, the host response, or a combination of both, Vero cells were infected at a high MOI to examine single-step growth (see Fig. S1 in the supplemental material). Following infection, both WT and dORF3-5 mutant virus grew to equivalent titers 24 h postinfection. In contrast, Calu-3 cells infected at a high MOI demonstrated robust attenuation of the dORF3-5 mutant beginning at 12 h and continuing over the course of the infection (Fig. 1C). Combined with the Vero cell results, the data suggest that host response limits the dORF3-5 mutant and confirm a critical role for the MERS-CoV accessory ORFs in successful infection of human respiratory cells.

10.1128/mBio.00665-17.1 FIG S1  No deficit in the dORF3-5 mutant in a single-step Vero cell growth curve. Download FIG S1, DOCX file, 0.1 MB. Copyright © 2017 Menachery et al. 2017 Menachery et al. This content is distributed under the terms of the Creative Commons Attribution 4.0 International license.

To examine changes in host responses, RNA expression was evaluated by microarray, with analysis revealing 5,046 differentially expressed genes between the dORF3-5 mutant and the WT. Using hierarchical clustering, differentially expressed genes were grouped into clusters based on their behaviors. This resulted in clusters of augmented and/or diminished genes, with the greatest changes observed 24 h postinfection (Fig. 1D). For clusters with increased expression in the mutant virus (clusters 1 to 3), functional categories were associated with disruption of normal cell host processes, including transcription regulation, protein transport, protein catabolism, chromatin modification, zinc binding, and more. Similarly, clusters generally associated with downregulation (Fig. 1D, clusters 4 to 6) include disruption of important cell function, including RNA splicing, protein complex assembly, and chromatin organization. We next performed a detailed enrichment analysis of gene changes between WT and mutant virus at 24 h postinfection. Differentially expressed genes were divided into several behavioral categories based on comparisons between mock-infected cells and cells infected with the wild type or mutant (e.g., DDU [“down up”] refers to a gene that is downregulated in the wild type compared to mock infection, downregulated in the mutant compared to mock infection, and upregulated in the mutant compared to the wild type). Using the DAVID online resource (https://david.ncifcrf.gov), genes from each behavioral category were used for functional enrichment analysis, thus showing what pathways likely displayed each behavior (see Tables S1 and S2 in the supplemental material). To simplify the results, DAVID groups similar enrichment hits (pathways/processes) into clusters. Accordingly, in Tables S1 and S2 each cluster of enrichment hits is represented only by the highest-scoring member of that cluster. Based on the behavioral categories and functional enrichment results, it can be seen that the dORF3-5 mutant upregulates response to virus (9.71) and defense responses (3.55) beyond the upregulation of the wild-type and upregulates genes related to ubiquitin conjugation (11.67) and zinc finger pathways (30.62) that were not upregulated in the wild type. Together, the modeling results indicate that infection with the dORF3-5 mutant stimulates a strong host response that disrupts normal cell function and host immunity and likely influences the overall outcome of infection.

10.1128/mBio.00665-17.2 TABLE S1  Comparative analysis of gene categories upregulated in the dORF3-5 mutant relative to wild-type MERS-CoV. Download TABLE S1, DOCX file, 0.8 MB. Copyright © 2017 Menachery et al. 2017 Menachery et al. This content is distributed under the terms of the Creative Commons Attribution 4.0 International license.

10.1128/mBio.00665-17.3 TABLE S2  Comparative analysis of gene categories downregulated in the dORF3-5 mutant relative to wild-type MERS-CoV. Download TABLE S2, DOCX file, 0.4 MB. Copyright © 2017 Menachery et al. 2017 Menachery et al. This content is distributed under the terms of the Creative Commons Attribution 4.0 International license.

### dORF3-5 mutant induces strong interferon responses.

**FIG 2:**
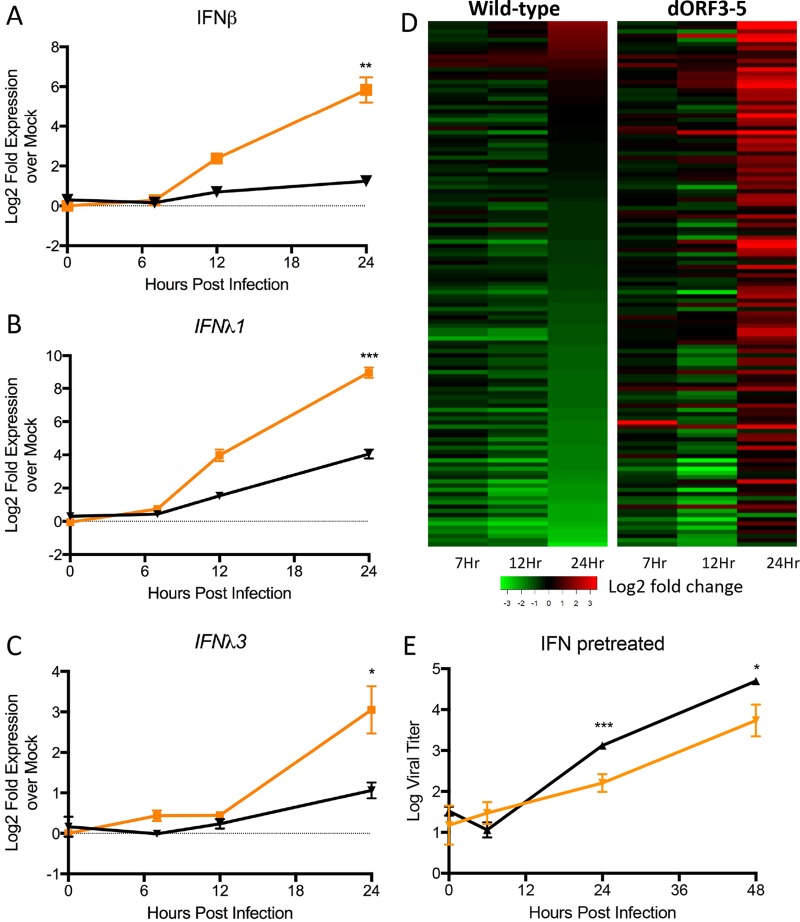
dORF3-5 mutant induces augmented IFN responses. (A to C) RNA expression of IFN-β (A), IFN-λ1 (B), and IFN-λ3 (C) following infection of Calu-3 2B4 cells with WT MERS-CoV (black) or the dORF3-5 mutant (orange) at an MOI of 5. (D) Consensus interferon-stimulated gene expression (log_2_ fold expression relative to mock infection) following wild-type or dORF3-5 mutant infection. (E) Vero cells treated with type I IFN (1,000 U) 16 h prior to infection with either WT MERS-CoV (black) or the dORF3-5 mutant (orange). *P* values are representative of Student’s *t* test: *, *P* < 0.05; **, *P* < 0.01; ***, *P* <0.001.

Based on strong attenuation and robust induction of host defense networks in Calu-3 cells, one possibility is that the dORF3-5 mutant induces a more robust interferon (IFN) response than WT virus. To examine IFN responses, RNA expression was evaluated following infection by microarray (Fig. 2A to C). The results reveal robust augmentation of IFN-β and IFN-λ in the mutant virus compared to the control. While responses were initially equal at early time points, the dORF3-5 mutant produces more IFN-β and IFN-λ transcript beginning at 12 h postinfection and increasing at 24 h; in contrast, WT MERS-CoV produces considerably less IFN-β and IFN-λ message. Notably, neither WT nor mutant virus induced increased expression of any IFN-α tested (data not shown). Extending our analysis, downstream interferon-stimulated genes (ISGs) were also examined. Using a previously established list of consensus Calu-3 ISGs (16), the dORF3-5 mutant was found to produce augmented expression of the vast majority, especially 24 h postinfection (Fig. 2D). While wild-type MERS-CoV expression produced subsets of augmented and downregulated genes relative to mock infection, the dORF3-5 mutant produced robust induction in the majority of ISGs. Extending these results, we examined the interferon sensitivity of the dORF3-5 mutant (Fig. 2E). While the dORF3-5 mutant has reduced replication relative to the control, type I IFN treatment does not ablate viral replication, suggesting the increased magnitude of ISGs rather than a specific ISG activity mediates dORF3-5 attenuation. Together, these results suggest that the absence of MERS accessory ORFs results in augmented stimulation of the IFN-β and IFN-λ response.

### Augmented NF-κB and inflammation stimulation in the dORF3-5 mutant.

**FIG 3:**
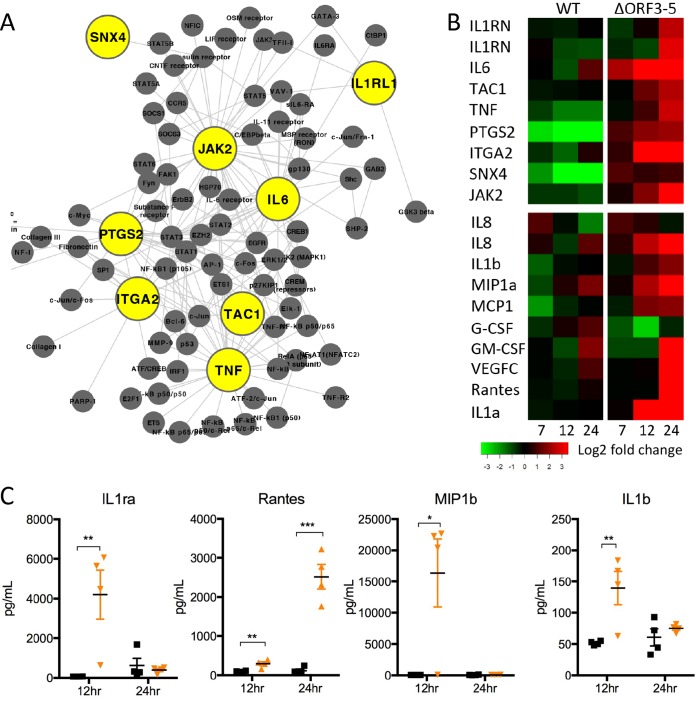
dORF3-5 mutant induces NF-κB-mediated inflammation. (A) Key gene regulators (yellow) and connections (gray) were identified by association with “positive regulation of inflammatory process,” representing genes that were upregulated at both 12 and 24 h in the dORF3-5 mutant virus relative to WT. (B) Expression of inflammatory regulators (top panel) and cytokines (bottom panel, log_2_ fold expression relative to mock infected) following WT (left) and dORF3-5 mutant infection. (C) Production of inflammatory cytokines following infection with WT (black) or dORF3-5 mutant (orange) virus 12 and 24 h postinfection of Calu-3 2B4 cells. *P* values are representative of Student’s *t* test: *, *P* < 0.05; **, *P* < 0.01; ***, *P* < 0.001.

In addition to robust IFN response, differential expression (DE) between mutant and wild-type virus showed statistical enrichment of inflammatory genes in the dORF3-5 mutant. *IL6*, *PTGS2*, and *TNF* and several other inflammation regulatory genes were found central to the network of genes activated in the mutant virus (Fig. 3A). Examination of regulator expression (Fig. 3B, top panel) shows clear stimulation in the mutant that is absent in the wild-type MERS-CoV infection. Extending this analysis to NF-κB-dependent cytokines and chemokines, the RNA expression data provides a clear signature of augmented inflammation in the dORF3-5 mutant. To verify this augmented inflammation response, protein analysis was conducted on cell supernatants 12 and 24 h postinfection (Fig. 3C). The results show robust induction of inflammatory cytokines and chemokines at 12 h postinfection, including interleukin-1 receptor antagonist (IL-1Ra), IL-1β, RANTES, and macrophage inflammatory protein 1α (MIP-1α). Overall, the results indicate that the dORF3-5 mutant prompts a robust inflammatory response in addition to a strong interferon response.

### dORF3-5 mutant attenuated *in vivo*.

**FIG 4:**
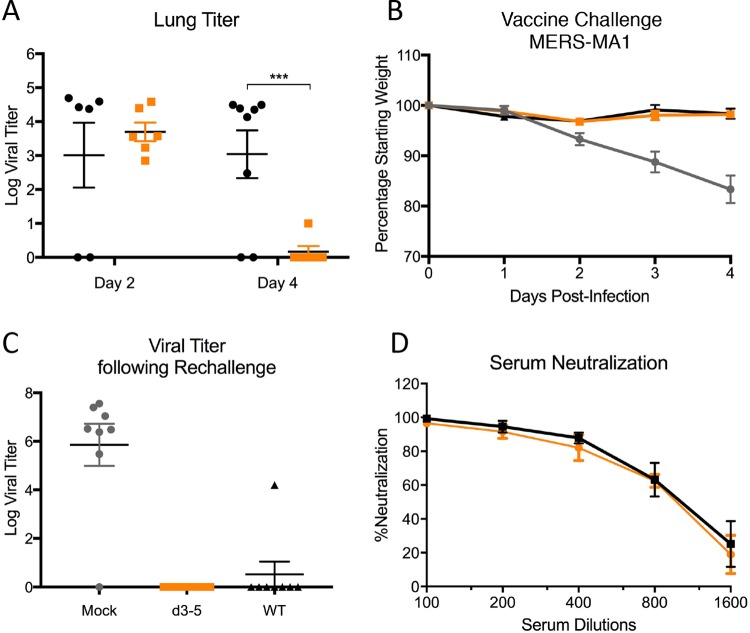
dORF3-5 mutation attenuates *in vivo* replication and protects from lethal challenge. (A) Lung titer from CRISPR-generated 288-330^+/+^ mice infected with WT (black) or dORF3-5 mutant (orange) virus at days 2 and 4. (B and C) Weight loss (B) and lung titer (C) following infection of mice vaccinated with WT (black) or dORF3-5 mutant (orange) virus or mock infected (gray). (D) Plaque reduction neutralization with sera from mice vaccinated with the WT (black) or dORF3-5 mutant (orange). The *P* value is representative of Student’s *t* test: ***, *P* < 0.001.

Having demonstrated replication attenuation as well as stimulation of both IFN and inflammation responses *in vitro*, the dORF3-5 mutant was evaluated *in vivo* utilizing a newly developed mouse model for MERS-CoV (22). Using a clustered regularly interspaced short palindromic repeat (CRISPR)-generated mouse targeting positions 288 and 330 of *Dpp4* (288-330^+/+^), the dORF3-5 mutant replicated less efficiently in the lung, but only at day 4 postinfection (Fig. 4A). While no weight loss was observed in any of these models because of the wild-type genome backbone (22), the results confirm *in vivo* attenuation of the dORF3-5 mutant. Importantly, despite replication attenuation, the dORF3-5 mutant was also capable of protection from a lethal MERS-CoV challenge. Following vaccination with the wild type or dORF3-5 mutant or mock infection, 288-330^+/+^ mice were challenged with a lethal dose of a mouse-adapted strain of MERS-CoV 15 (Fig. 4B and C) (22). While mock-infected mice experienced rapid weight loss through day 4, both wild-type- and dORF3-5-vaccinated mice were protected from weight loss (Fig. 4B) and viral replication (Fig. 4C). Notably, 50% plaque reduction neutralization test (PRNT_50_) titers showed robust antibody neutralization with wild-type and dORF3-5 mutant vaccination (Fig. 4D), suggesting that replication attenuation has no significant impact on the generation of a protective immune response. Together, the data indicate that the MERS dORF3-5 mutant has some potential as a live attenuated vaccine candidate.

### dORF3-5 mutant attenuated in mouse-adapted backbone.

**FIG 5:**
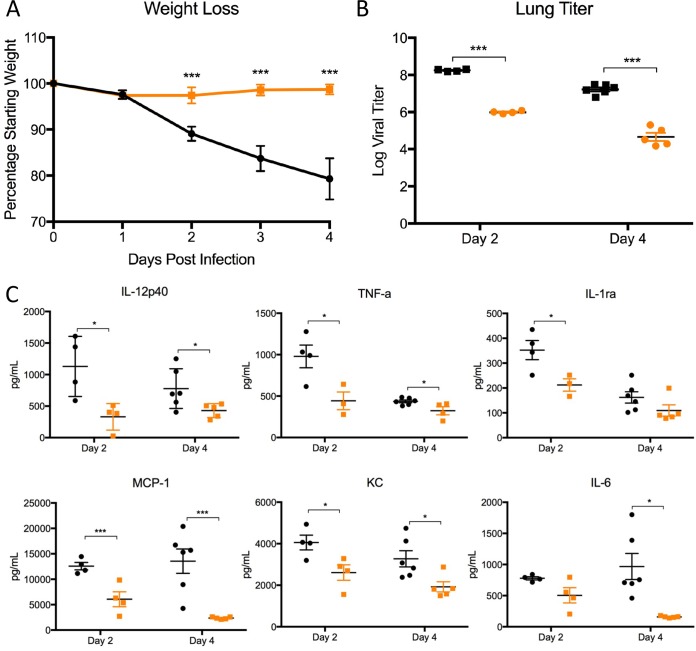
dORF3-5 mutation attenuates the virulent MERS-CoV strain. (A and B) Weight loss (A) and lung titers (B) following infection of CRISPR-generated 288-330^+/+^ mice infected with MERS-CoV MA1 (black) or dORF3-5 MA1 (orange) at days 2 and 4. (C) Production of inflammatory cytokines following infection with mouse-adapted WT (black) or dORF3-5 mutant (orange) virus 2 or 4 days postinfection. *P* values are representative of Student’s *t* test: *, *P* < 0.05; ***, *P* <0.001.

While replication attenuation was observed in the wild-type MERS-CoV backbone, it remained possible that the dORF3-5 mutant may not be sufficiently attenuated in a virulent backbone. To test this question, the dORF3-5 mutant was generated in the context of a mouse-adapted infectious clone. Derived from a plaque-purified isolate of MERS-CoV 15, the MERS-C2 clone had several mutations in its backbone, including point mutations in nonstructural protein 2 (NSP2), NSP6, and NSP8 as well as a large deletion of a portion of ORF4b (22). The removal of an accessory ORF in this and other mouse-adapted clones raised concerns that accessory ORF mutation may augment pathogenesis. However, following infection with the dORF3-5 mutant in the mouse-adapted backbone, 288-330^+/+^ mice showed no pathogenesis in terms of weight loss (Fig. 5A). Similarly, viral replication of the mutant virus is attenuated at both days 2 and 4 postinfection (Fig. 5B). Notably, examination of the cytokine response in the lung indicated attenuated inflammatory responses following infection with the dORF3-5 mutant relative to the mouse-adapted control virus (Fig. 5C); the largest differences were noted at day 2, suggesting that dORF3-5 attenuation occurs early during *in vivo* infection. Together, the results illustrate clear attenuation of the dORF3-5 mutant in the context of a virulent backbone and signal its possible utility as a live attenuated vaccine platform.

### ORF5 plays a role in limiting inflammation.

**FIG 6:**
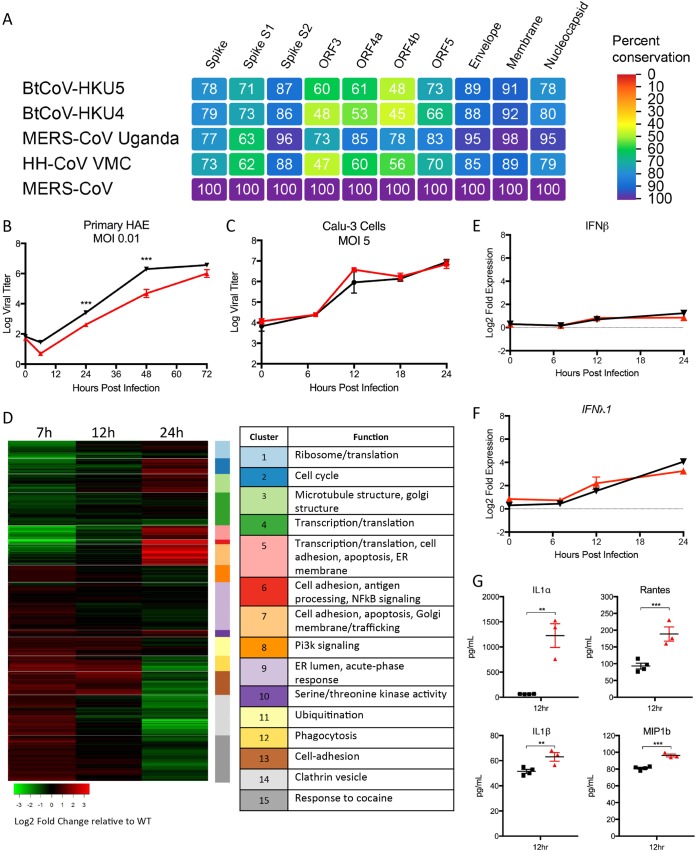
ORF5 plays role in modulation of NF-κB-mediated inflammation. (A) Heat maps were constructed from a set of group 2C coronaviruses, including consensus bat HKU4 (BtCoV-HKU4), consensus bat HKU5 (BtCoV-HKU5), MERS-CoV Uganda (PREDICT/PDF-2180 [3]), and hedgehog (HH) CoV VMC (ErinaceusCoV [42]), using alignment data paired with neighbor-joining phylogenetic trees built in Geneious (v.9.1.5) and visualized in EvolView (EvolGenius). The trees show the degree of genetic similarity of MERS-CoV ORFs across group 2C. (B) Primary human airway epithelial cells infected with WT (black) or dORF5 mutant virus at an MOI of 0.01. (B to F) Calu-3 2B4 cells infected with WT (black) or dORF5 mutant virus at an MOI of 5. (C and D) Viral replication (C) and RNA expression analysis (D) of dORF5 mutant relative to wild-type virus infection. Clusters were defined by functional category and identified by differential expression score. (E and F) RNA expression of IFN-β (E) and IFN-λ1 (F) following infection. (G) Production of inflammatory cytokines following infection with WT (black) or dORF5 mutant (red) virus 12 h postinfection of Calu-3 2B4 cells. *P* values are representative of Student’s *t* test: **, *P* < 0.01; ***, *P* < 0.001.

Having fully established dORF3-5 attenuation *in vitro* and *in vivo*, we next examined the role of the individual ORFs in contribution to attenuation. As expected, the accessory ORFs have the greatest diversity even within the group (Fig. 6A). For 2C CoVs which include MERS-CoV, the greatest amino acid diversity is observed in ORF3, -4a, -4b, and -5, suggesting that evolutionary pressures have shaped proteins, likely to adapt to specific aspects of their host species. Several reports have highlighted both ORF4a and -4b as IFN antagonists as well as disrupting elements of the interferon-stimulated gene response (18, 23, 24). In contrast, little characterization of dORF5 has occurred in the context of MERS-CoV infection. With this in mind, we utilized a previously described MERS mutant that replaces ORF5 with red fluorescent protein (RFP) in order to characterize the host response in its absence. Following infection of primary HAE cells, the dORF5 mutant had a modest, but significant attenuation in replication relative to WT virus 24 and 48 h postinfection; by 72 h, the dORF5 mutant had nearly equivalent titers (Fig. 6B). Similar to Vero cells (20), the dORF5 mutant had no significant replication attenuation in Calu-3 cells following high-MOI infection (Fig. 6C). However, broad changes in the host response were observed in RNA expression (Fig. 6D); upon examination of expression relative to wild-type virus, the dORF5 mutant had more modest changes than the dORF3-5 mutant (Fig. 1). However, several pathways associated with apoptosis, NF-κB signaling, and serine/threonine kinase activity were augmented in the dORF5 mutant (clusters 5, 6, 7, and 10). Notably, the dORF5 mutant failed to induce robust type I and type III IFN responses, suggesting no specific role in IFN antagonism (Fig. 6E and F). In contrast, inflammatory cytokines demonstrated augmented production following dORF5 infection relative to the WT (Fig. 6G). While this stimulation pales compared to that of the dORF3-5 mutant, the data argue for a role in dORF5 in disrupting elements of NF-κB activation and the host inflammation response following MERS-CoV infection.

## DISCUSSION

Utilizing the MERS-CoV infectious clone, this study underscores the importance of MERS accessory ORFs in both infection and pathogenesis. Our results indicate that attenuation of the MERS dORF3-5 mutants is primarily driven by augmented host responses rather than a defect in aspects of viral replication alone. Disruption of normal cell processes, increased magnitude of IFN-β and IFN-λ responses, and stimulation of robust inflammation produce replication attenuation of the dORF3-5 mutant in immunocompetent Calu-3 and human airway epithelial cells. In addition, the loss of accessory ORFs reduces both replication and pathogenesis *in vivo*. Notably, the combination of viral replication attenuation and augmented immune responses opened the possibility of using the dORF3-5 mutant as a live attenuated vaccine platform. With minimal signs of pathogenesis, the dORF3-5 vaccine provided complete protection from lethal MERS-CoV challenge and robust viral neutralization. Importantly, the absence of ORF5 is implicated in augmented inflammation responses, although the precise biochemical mechanisms of antagonism will require additional study. Together, the results demonstrate the importance of MERS-CoV accessory ORFs in viral infection and may provide a platform for developing therapeutics for future CoV outbreak strains by targeting accessory ORF functions.

With high variability across the CoV family, accessory ORFs are not required for replication, but often perform important functions within the context of infection. The majority of characterized CoV accessory ORFs have been implicated in antagonizing the host response (25). Not surprisingly, MERS-CoV ORF4a and -4b have been previously identified for their roles in modulation of type I IFN responses (18, 23, 24, 26). With removal of these viral proteins from the dORF3-5 mutant, stimulation of robust type I and III IFN responses was not unexpected, and downstream ISG networks strongly support the biochemical functions defined for ORF4a and ORF4b. In contrast, induction of NF-κB-based inflammation suggests that MERS accessory ORFs may also modulate other aspects of host immunity. While previous work has implicated ORF4a in induction of stress granules (18), the removal of MERS ORF5 also activated inflammatory gene clusters and produced a robust inflammatory cytokine cascade following infection. However, inflammation induction in the ORF5 deletion virus paled relative to that in the dORF3-5 mutant, suggesting ORF5 may only partially influence the inflammatory response and may work in concert with ORF4a. Importantly, the dORF5 mutant failed to induce changes in IFN pathways indicating a limited role for the accessory ORF. Overexpression studies offer an avenue to decipher ORF5 mechanisms by biochemical assays on known inflammatory pathways (27); alternatively, new approaches like tagged protein/mass spectrometry provide a rapid means to identify interaction partners and derive key insights (28, 29). Overall, the data suggest that MERS accessory ORFs target multiple aspects of the host immune response and play a critical role in infection and pathogenesis.

Shaped by evolutionary pressures, CoV accessory proteins are often important tools for modulating aspect of host immunity, including IFN stimulation, inflammation, and cell cycle arrest (25). However, maintenance of these functions may occur by a number of different mechanisms leading to diversity in accessory ORF proteins and reduced sequence conservation. Group 2C CoVs model this pattern with low conservation of ORF3, -4a, -4b, and -5 amino acid identity relative to other CoV proteins. These results leave the possibility that functions may be tailored for individual reservoir hosts and result in variation even within highly related coronaviruses. While previous work has indicated conservation of ORF4b function, overall efficiency and target variation suggest modest but important differences across group 2C CoVs (19). Exchange of accessory ORFs across strains may have important implications for infection and pathogenesis. In addition, species changes may also alter efficacy and function. For example, MERS ORF5 likely modulates NF-κB-mediated inflammation and may be key to persistence in bat or camel species. However, ORF5 has been deleted in mouse-adapted MERS-CoV strains (22), and the effects suggest its absence may augment pathogenesis in mice. Notably, deletion and truncation in ORF5 like other accessory ORFs in both MERS-CoV and SARS-CoV suggest that ineffective or unnecessary ORFs may be discarded (30–32). Alternatively, the acquisition of novel accessory ORF function may permit emergence in a new host. In both situations, the resulting viruses may be more pathogenic, but potentially limited in other ways, including spread and persistence. Overall, the data indicate further study and surveillance of accessory ORFs in zoonotic CoV strains is needed going forward.

While several MERS-CoV vaccine approaches have been described (33), studies have been limited by the absence of robust animal models that recapitulate physiologically relevant disease. Similar to SARS-CoV accessory ORF mutants (34), the dORF3-5 mutant in the wild-type MERS-CoV backbone demonstrated replication attenuation in several *in vivo* models and protected from lethal challenge with a mouse-adapted strain (22). Importantly, the deletion of ORF3-5 also attenuated the virulent mouse-adapted MERS-CoV indicating a role for the MERS accessory ORFs in lethal disease. Notably, augmented inflammation observed during *in vitro* infection with the dORF3-5 mutant is inverted *in vivo* (Fig. 5C). These discordant results suggest a rapid, transient inflammation may occur following dORF3-5 infection and may result in more rapid clearance and/or augmented immunity. Together, the *in vivo* data indicate that the disruption of accessory ORFs in parallel may provide a suitable and effective rapid response vaccine platform for future emergent CoVs.

While less appealing than subunit and inactivated-virus approaches, live attenuated approaches for MERS-CoV must also be considered in the context of the failure of SARS vaccines (35). While effective in young animals (36), testing in aged animal models and following heterologous challenge are key metrics that identified deficits in SARS-CoV vaccines, including vaccine-induced inflammation and eosinophilia (35, 37). Considering the augmented inflammation observed in the mutant *in vitro*, both aged and heterologous challenge models provide important metrics for vaccine safety that are difficult to test in the context of homologous challenge. In addition, the validity of dORF3-5 as a vaccine platform requires examination of genetic stability. Initial studies need to determine if sterilizing immunity is established following dORF3-5 vaccine challenge at earlier times. Low-level replication permits the introduction of compensatory mutations; these mutations may restore virulence, and this is a significant concern for all live attenuated virus strains, as seen previously in CoV deletion viruses (38). While tissue culture passage of the dORF3-5 mutant suggests no restoration of viral fitness, further stability studies in immunodeficient mice as well as mouse passage are required to advance the dORF3-5 mutant as a live attenuated vaccine platform (39).

However, for MERS-CoV, the increased magnitude of both IFN and inflammation by the dORF3-5 mutant provides an advantage with the induction of a potent immune response despite replication attenuation. The augmented response may produce improved vaccine efficacy both in aged models and in heterologous challenge. Importantly, based on critical roles for accessory proteins in SARS, MERS, and other CoV infections, targeted deletions of these nonessential ORFs may provide a universal platform for targeting future emergent strains. With the ongoing identification of novel MERS- and SARS-like viruses in zoonotic populations, disruption of accessory ORFs in parallel may provide a suitable and effective rapid response platform.

Overall, this article describes the importance of accessory protein in the context of MERS-CoV infection and pathogenesis. The attenuation of the dORF3-5 mutant is primarily a product of induction of differential host responses, including both IFN and inflammation pathways. Thus, loss of the accessory ORFs attenuates viral replication *in vitro* and pathogenesis *in vivo*. Importantly, these deficits can be leveraged in the context of a live attenuated MERS-CoV platform. While IFN induction has been previously been linked to both ORF4a and ORF4b, our results indicate a role for ORF5 in modulation of NF-κB-mediated inflammation. Together, the data provide clear evidence for MERS-CoV accessory ORFs as key tools needed for infection and pathogenesis.

## MATERIALS AND METHODS

### Cells and viruses.

Wild-type and mutant MERS-CoVs were previously described (20) and cultured on Vero 81 cells, grown in Dulbecco’s modified Eagle’s medium (DMEM) (Gibco, CA) and 5% fetal clone serum (HyClone, South Logan, UT) along with an antibiotic/antimycotic (Gibco, Carlsbad, CA). Growth curves in Vero, Calu-3 2B4, and human airway epithelial (HAE) cells were performed as previously described (9, 30). Briefly, cells were washed with phosphate-buffered saline (PBS) and inoculated with virus or mock diluted in PBS for 40 min at 37°C. Following inoculation, cells were washed 3 times, and fresh medium was added to signify time zero. Samples were harvested at the described time point. For IFN pretreatments, 100 U/ml of recombinant human IFN-β (PBL Laboratories) was added to cells 16 h prior to inoculation, and cells were infected as described above. All virus cultivation was performed in a biosafety level 3 (BSL3) laboratory with redundant fans in biosafety cabinets as described previously by our group (31, 32). All personnel wore a 3M Breathe Easy powered air-purifying respirator with Tyvek suits, aprons, and booties and were double gloved.

### Construction of wild-type and mutant viruses.

Both wild-type and mutant viruses were derived from either the MERS-CoV wild type or the corresponding mouse-adapted (MA1 [herein referred to as WT]) infectious clone as previously described. Generation of the dORF3-5 and dORF5 mutants was previously described.

### RNA isolation, microarray processing, and identification of DE.

RNA isolation and microarray processing from Calu-3 cells were carried out as previously described (40). Differential expression (DE) was determined by comparing virus-infected replicates to time-matched mock replicates. Criteria for DE in determining the consensus ISG list were an absolute log_2_ fold change of >1.5 and a false discovery rate (FDR)-adjusted *P* value of <0.05 for a given time point.

### Clustering and functional enrichment.

Genes identified as differentially expressed were used to generate clustered expression heat maps. Hierarchical clustering (using Euclidean distance and complete linkage clustering) was used to cluster gene expression according to behavior across experimental conditions. The DAVID online resource (https://david.ncifcrf.gov) was used to acquire functional enrichment results for the genes in each cluster. DAVID output was manually summarized for each cluster. Plots were generated with R.

For the analysis in Tables S1 and S2, three comparisons were used to group genes into distinct behavioral categories: wild-type virus compared to mock infected, mutant virus compared to mock infected, and mutant virus compared to wild type, all at the 24-h time point. Genes were assigned a behavioral category based on whether they were up- or downregulated in each of these three comparisons. The genes in each category were then submitted to DAVID analysis, which resulted in clusters of functional enrichment hits grouped according to gene set overlap (accession no. 17784955). To simplify the results, each cluster is represented only by the highest-scoring enrichment hit in each cluster and scored according to the negative log_10_ *P* value of the significance of the enrichment for that top hit.

### Ethics statement.

This study was carried out in accordance with the recommendations for care and use of animals by the Office of Laboratory Animal Welfare (OLAW), National Institutes of Health. The Institutional Animal Care and Use Committee of the University of North Carolina at Chapel Hill (IACUC, UNC [permit no. A-3410-01]) approved the animal study protocol (IACUC protocol no. 15-009 and 13-072) followed in this article.

### Mouse infections and vaccinations.

Ten- to 20-week old CRISPER-generated 288-330^+/+^ C57BL/6 mice were anesthetized with ketamine and xylazine (as per IACUC, UNC guidelines) and intranasally inoculated with a 50-µl volume containing 10^6^ PFU of MERS-CoV WT, SARS-CoV dORF3-5 virus, mouse-adapted variants, or the PBS mock infection control as indicated in the figure legends. Infected animals were monitored for weight loss, morbidity, and clinical signs of disease, and lung titers were determined as described previously (36). For vaccination experiments, 10- to 20-week-old 288-330^+/+^ mice were infected with 10^6^ PFU of dORF3-5 as described above, monitored for clinical symptoms for 7 days, and then challenged 4 weeks postvaccination with 10^6^ PFU MERS-CoV MA1-passaged virus. Animal housing, care, and experimental protocols were in accordance with IACUC, UNC guidelines.

### Accession number(s).

Raw microarray data for these studies were deposited in publicly available databases in the National Center for Biotechnology Information (NCBI) Gene Expression Omnibus (41) and are accessible through GEO accession no. GSE65574 (http://www.ncbi.nlm.nih.gov/geo/query/acc.cgi?acc=GSE65574).
