# Supplementary material for: MERS-CoV Accessory ORFs Play Key Role for Infection and Pathogenesis
Source: mBio. 2017 Aug 22;8(4):e00665-17. doi: 10.1128/mBio.00665-17 (PMC5565963; doi:10.1128/mBio.00665-17)
Supplement: FIG S1 [file mbo004173446sf1.docx]

**Supplemental Figure 1. No deficit in dORF3-5 mutant in single step VERO growth curve.** VERO cells were infected with wild-type MERS-CoV (Black bars) or the dORF3-5 mutant (Orange bars) at and MOI of 5. Viral replication was assayed at 6 and 24 hours post infection by plaque assay.
