# Supplementary material for: MERS-CoV Accessory ORFs Play Key Role for Infection and Pathogenesis
Source: mBio. 2017 Aug 22;8(4):e00665-17. doi: 10.1128/mBio.00665-17 (PMC5565963; doi:10.1128/mBio.00665-17)

**Supplementary Table 2. Comparative analysis of gene categories downregulated in dORF3-5 relative wild-type MERS-CoV.** Using DAVID analysis, genes with differential expression at 24 hours post infection were clustered in functional groups based on log2 fold expression and cluster significance. The gene clusters were further defined as U (upregulated) or D (downregulated) based on relative expression in three categories: 1) WT expression relative to mock. 2) dORF3-5 expression relative to mock. 3) dORF3-5 expression relative to WT. This table represents clusters with lower expression in dORF3-5 with scores >1.75.


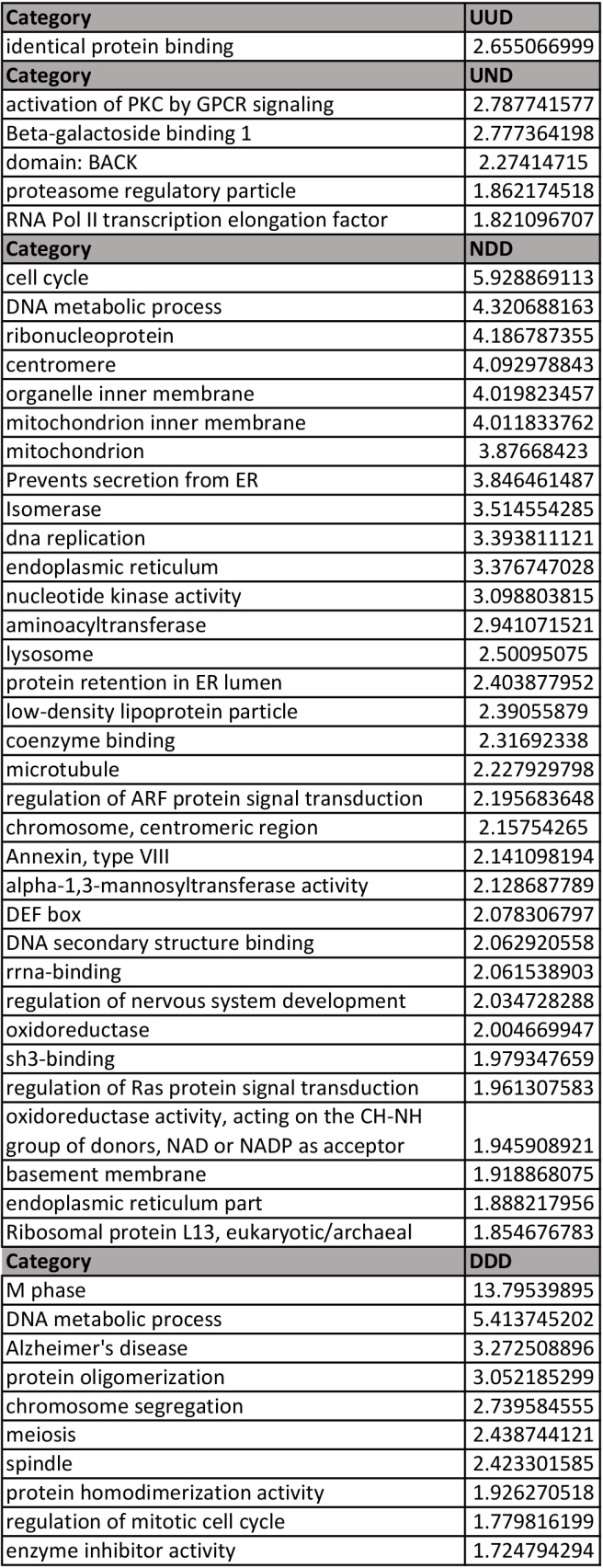

Supplement: TABLE S2 [file mbo004173446st2.docx]
